# Realism, Conservatism, and Tiered Ecological Risk Assessment

**DOI:** 10.3390/ecologies3020011

**Published:** 2022-05-26

**Authors:** Matthew A. Etterson

**Affiliations:** US Environmental Protection Agency, Office of Research and Development, Great Lakes Toxicology and Ecology Division, Duluth, MN 55804, USA

**Keywords:** MCnest, endogenous lifecycle model, population model, PopGUIDE, risk assessment

## Abstract

Recent research has provided valuable momentum for the development and use of population models for ecological risk assessment (ERA). In general, ERA proceeds along a tiered strategy, with conservative assumptions deployed at lower tiers that are relaxed at higher tiers with ever more realistic models. As the tier increases, so do the levels of time and effort required by the assessor. When faced with many stressors, species, and habitats, risk assessors need to find efficiencies. Conservative lower-tier approaches are well established, but higher-tier models often prioritize accuracy, and conservative approaches are relatively unexplored at higher tiers. A principle of efficiency for ecological modeling for population-level ecological risk assessment is articulated and evaluated against a conceptual model and an existing set of avian models for chemical risk assessment. Here, four published avian models are reviewed in increasing order of realism (risk quotient → Markov chain nest productivity model → endogenous lifecycle model → spatially explicit population model). Models are compared in a pairwise fashion according to increasing realism and evaluated as to whether conservatism increases or decreases with each step. The principle of efficiency is shown to be a challenging ideal, though some cause for optimism is identified. Strategies are suggested for studying efficiency in tiered ecological model deployment.

## 1. Introduction

Interest in applied population models has grown rapidly over the last half-century, driven in part by the utility of population models for conservation and management. Two distinct threads in applied population modeling have emerged, population viability analysis (PVA) and population-level risk assessment (PLRA). PVA models have long supported protection and management for the recovery of vulnerable, threatened, and endangered species [1]. In contrast, regulatory acceptance of PLRA models has been slow [2–4], though demonstrations and reviews of PLRA models have been available for decades [5–14].

The primary objective of PLRA is to evaluate the potential for adverse effects of environmental contaminants on populations resulting from effects on exposed individuals [15]. As the principles of ecological risk assessment (ERA) developed to embrace a tiered evaluation strategy, population models were recognized as a valuable tool for higher-tier risk assessment when screening assessments suggested potential risk [16]. PopGUIDE [3] and associated works [4,12] have provided a roadmap for the development of population models for PLRA that considers the regulatory framework under which the risk assessment is conducted, the availability of organismal, toxicological, and exposure data, and the resources available for model development [3–14,17].

In the US Environmental Protection Agency (USEPA)’s tiered process for ERA, lower tiers are typically designed to be more conservative [18] so that chemicals and use patterns with low risk can be quickly triaged. For example, the USEPA’s Office of Pesticide Programs compares risk quotients (RQ = Exposure/Toxicity, a more precise definition is provided below) to levels of concern (LOC), where escalation to higher tiers may be required if RQ > LOC and additional information is needed to better understand risk [16,19]. Exposure/Toxicity evaluations may be made intentionally conservative by using exposure estimates from the upper tails of measured or modeled exposure distributions [19], by setting low LOCs, or by choosing toxicity endpoints from the lower tails of measured toxicity values [20]. In those cases, when Exposure/Toxicity < LOC, then we have confidence that the risk is truly low. This example also highlights the important role of parameterization (in this case, choice of specific exposure, toxicity, or LOC value for the RQ) in determining whether a model prediction is conservative. Because RQs so designed are conservative, RQ > LOC does not necessarily mean that the risk is unacceptable. Thus, an important function of tier escalation is to progressively relax conservative assumptions to obtain a more refined understanding of risk.

A conservative model prediction is one that overestimates the true magnitude of effect for a given risk scenario. It follows then that conservative model predictions are those that are positively biased (bias > 0), where bias is defined in the usual way (Equation (1)) as the expected value of the difference between the predicted effect magnitude and the true effect magnitude.


(1) $$bias\left(\hat{y}\right)=E\left(\hat{y}-y\right)$$

In Equation (1), y represents effect magnitudes (risk quotient, changes in fecundity, fitness, population growth rate, etc.). The term $\hat{y}$ represents model predicted effect magnitude, whereas y represents the ‘true’ (unknown) effect magnitude. In practice, for the discussions that follow, these would need to be scaled appropriately to be comparable across tiers. These and other complications are illustrated and discussed below.

In PLRA, tier escalation is also associated with increased model complexity and realism with the goal of reducing uncertainty [3]. Together, these principles require a *designed* inverse relationship between model complexity and positive bias with tier escalation. If the relationship is so designed, then a determination of “low risk” at any tier is sufficient justification for terminating the escalation. Time and effort on the part of the risk assessor also increase with tier escalation so that early identification of “low risk” scenarios is a more efficient use of time and resources. Ideally, then, a subordinate tier produces a determination of “low risk”, or the ultimate tier converges on an accurate and unbiased representation of risk. If this relationship does not hold, then the presumption of safety conferred by passing a tier may be flawed and may not justify terminating the assessment.

The above arguments can be summarized into an efficiency principle for PLRA:

If an exposure scenario represents low risk for a given species, we would like to make a “low risk” determination at the earliest possible tier and using the simplest possible model(s).

In this sense, the “simplest possible model” is the first model in the tier escalation sequence that renders a “low/no risk” determination. A similar argument could be made for quickly identifying exposure scenarios that pose a clear risk, but this is not considered further herein. The resulting vision is of a series of increasingly realistic models that progressively decrease uncertainty while also reducing positive bias in model predictions of effect magnitude by relaxing conservative assumptions. This principle is articulated based on personal observation of how tiered ecological risk assessment seems to be practiced and/or envisioned.

The efficiency principle articulated above may conflict with generally accepted practices for the development and deployment of ecological models, which will be referred to collectively as “best practices” [3–14,17,21]. Under best practices, parsimony is applied to optimize the complexity of a particular model given the available data and the objectives of the risk assessment. In the contrasting context considered here, the risk assessor has a sequence of previously developed models of increasing complexity in his or her toolbox. That sequence is an efficient sequence if model predictions of effect magnitude are positively biased and that positive bias decreases with increasing complexity and increasing realism. With an efficient model sequence, a no-risk determination at any point strongly suggests a no-risk determination at higher tiers, thus justifying terminating the assessment. The point of complexity (tier) at which a no-risk determination occurs will differ depending on the context of the risk assessment and should not occur at all if the true risk is unacceptable.

Much recent literature has been devoted to trying to understand why higher-dimensional, more realistic, ecological models, such as population models, are not used more routinely in ecological risk assessment. In this paper, it is hypothesized that the principle of efficiency, articulated above, is inconsistent with best practices for ecological model development that focus on model accuracy and on fitting models commensurate with available data [21]. In short, we do not yet know how to identify and deploy a decreasingly conservative set of off-ramps that would allow risk assessors to escalate along a model sequence only so far as is necessary for a risk decision. In the following, I first develop a conceptual model for comparing the performance of the efficiency principle to an ideal unbiased model sequence. Following conceptual model development and analysis, I critically evaluate my own past work and the extent to which it could satisfy the efficiency principle. In the model review, I focus on my own work for three reasons: (1) I am most familiar with it and the assumptions made during development and application; (2) these models are likely candidates from which the EPA could choose when defining an escalation sequence for avian PLRA; and (3) these models form a loosely nested sequence, with output at tier n-1 serving as input to tier n, thus guaranteeing increased model complexity along the sequence. The reviewed models were not necessarily developed for this purpose, which complicates the transitions to higher complexity, but as noted above, this is likely to be the general case. My primary objective is to illustrate the conflict between the efficiency principle and best practices for model development and the difficulties we will face in reconciling this conflict.

## 2. Materials and Methods

### 2.1. Conceptual Model

A conceptual model is developed to illustrate anticipated changes in model-predicted effect magnitudes with tier escalation in ecological risk assessment. The model must satisfy the following criteria. Under the efficiency principle, positive bias should decline with increasing tier, increasing realism, and decreasing uncertainty towards an accurate prediction of the true magnitude of effect. Under best practices, models are assumed unbiased, and uncertainty around model predictions declines with increasing tier. In either case, a risk scenario should be discovered to be safe at any given tier when the predicted effect magnitude is lower than the maximum acceptable effect magnitude.

Following conceptual model development and analysis, a series of models with escalating complexity and realism is reviewed, evaluating at each step conditions under which the next higher model includes processes and data that would likely make its predictions more or less conservative than its predecessor. In each case, models are nested within one another (or can be conceptualized that way after the fact), which increases comparability across tiers and creates a strictly increasing sequence of model complexity. Where possible, comparisons are illustrated with previously published parameterizations of each model, though some novel parameterizations are used. Models are deployed heuristically and in keeping with their design, but no attempt is made to verify or validate their predictions, which is outside the scope of this exercise. The four models considered for an escalation sequence are given in Table 1, which yields three escalation steps. Some background on each model is given below, with key references for further details.

### 2.2. Risk Quotients

Risk quotients (RQ) are ratios of expected exposure to a measured toxicity endpoint, where the numerator and denominator are expressed in the same units (e.g., mg chemical/kg body weight). For avian RQs, toxicity endpoints are the median lethal dose (LD50) from an avian acute oral toxicity study [26], the median lethal concentration (LC50) from an avian dietary toxicity study [27], or No Observed Adverse Effects Levels (NOAELs) from an avian reproduction test [28]. RQs are evaluated by comparing to Levels of Concern (LOC), which are 0.5 for the LD50 and LC50 RQs and 1 for RQs from the reproduction test. As noted above, RQs are designed to be conservative so that RQ < LOC can be confidently interpreted as representing minimal or low risk.

### 2.3. MCnest

The Markov Chain Nest Productivity Model (MCnest) estimates the impact of pesticide-use scenarios on the seasonal productivity of bird populations. The primary objective of model development was to give risk assessors a way to make ecological inferences about avian reproduction from standard toxicity test results. MCnest integrates toxicity information from the same three standardized avian toxicity tests described above [26–28] with information on species life history and the timing of pesticide applications relative to the timing of avian breeding seasons. The model expands the RQ concept by comparing dynamic modeled exposure to surrogate toxicity endpoints [29] on a daily basis while the stochastic breeding model is running. Surrogate endpoints are designed to be phase-specific (e.g., egg-development, egg-laying, incubation, nestling care) and are chosen carefully from the suite of measured endpoints from the toxicity tests. A nest attempt is assumed to fail if the appropriate exposure measure exceeds the surrogate endpoint. Following nest completion, whether successful or failed, a female bird is assumed to renest according to typical species-specific propensities. The trajectory of success and failure is tracked for each female, and the total reproductive output (seasonal productivity) of each female is calculated over the breeding season. Typical usage is to compare seasonal productivity under alternative pesticide use scenarios, including control simulations with no pesticide usage.

MCnest incorporates two alternative models of avian pesticide exposure, the Terrestrial Residue Exposure model (T-REX) [19] and the Terrestrial Investigation Model (TIM) [30]. T-REX is a screening-level exposure model that estimates pesticide residues on classes of dietary items (seeds, fruit, invertebrates, foliage, and grass) following pesticide applications. T-REX inputs include date(s) of application, application rate (lbs/acre), and foliar dissipation half-life. These data are integrated with prior empirical data on the distribution of pesticide concentrations on dietary items following known application rates to calculate concentrations of pesticides in avian food [31,32]. TIM is a refined exposure model that also incorporates potential exposure through inhalation, dermal absorption, drinking water, and spray drift. TIM has many more input parameters, including information on field application methods, chemical properties (water solubility, partitioning coefficients between water and air, octanol, and organic carbon), toxicity, and species life history (diet, body weight, and foraging dynamics, including time of day and time spent on field). TIM exposure estimates from non-dietary routes are converted to dietary equivalents to estimate total exposure. Regardless of which exposure model is chosen and parameterized, MCnest compares the resulting exposure estimates to surrogate endpoints carefully chosen to be specific to different phases in the nest cycle [29,33].

### 2.4. Endogenous Lifecycle Models

Endogenous lifecycle models (ELMs) were recently proposed [24] as an intermediate step between individual-level models and population models. The primary objective in developing ELMs was to provide a robust modeling framework for predicting changes in individual fitness due to disruption of endogenous physiological processes, such as occur along adverse outcome pathways (AOP) [34]. ELMs are not population models though they superficially resemble density-independent matrix models. In comparison with MCnest, ELMs are more realistic and more complex because they include the full annual cycle rather than just the avian breeding season, which is typically limited to spring and early summer for north temperate birds. MCnest predictions may be used as an input parameter for ELMs, creating a nested model set and increasing comparability across tiers (in-depth example provided in [24]). ELMs fall short of population models because they do not predict population trajectories. Nor do they include the many exogenous stressors (except chemical exposure) to which individuals in a population may be exposed. Rather, an ELM predicts individual fitness on an annual or lifetime basis under alternative exposure conditions. The following two ELM fitness equations [24] are relevant to the subsequent material:

(2) $$LRS=f\frac{s_{j}}{1-s_{a}}$$

(3) $$\lambda_{f}=s_{a}+fs_{j}$$

Equation (2) gives the expected lifetime reproductive success (LRS) of a bird with a typical temperate passerine-like lifecycle. Equation (3) gives intrinsic fitness (λ_f_ = the expected annual production of genetic descendants, including self) for the same lifecycle. In Equations (2) and (3), s_a_ = annual adult survival after age 1, s_j_ = annual juvenile survival (before age 1) and f = annual number of offspring raised to fledging.

### 2.5. Spatially Explicit Population Model

A spatially explicit population model (SEPM) [25] for the California gnatcatcher (*Polioptila californica*) was created by implementing MCnest within HexSim [35] to evaluate the potential impacts of pesticide use on this federally threatened species. Resources (collectively “habitat quality”) were modeled using land cover and land use maps together with an existing logistic regression model [36]. Individual female reproductive success was modeled using MCnest, and pesticide usage was modeled using maps of agricultural land use within the gnatcatcher range. Individual lifecycles were modeled as location-specific ELMs, with MCnest fecundity predictions as inputs. Density dependence and movement limitation were generated as emergent properties of dispersal and carrying capacity determined by habitat quality. SEPMs are a further escalation of complexity and represent one of the most realistic ways to model resource limitation, movement constraints, and the population processes that emerge from these effects (e.g., density dependence) [37].

## 3. Results and Discussion

### 3.1. Conceptual Model & Analysis

**Figure 1.**
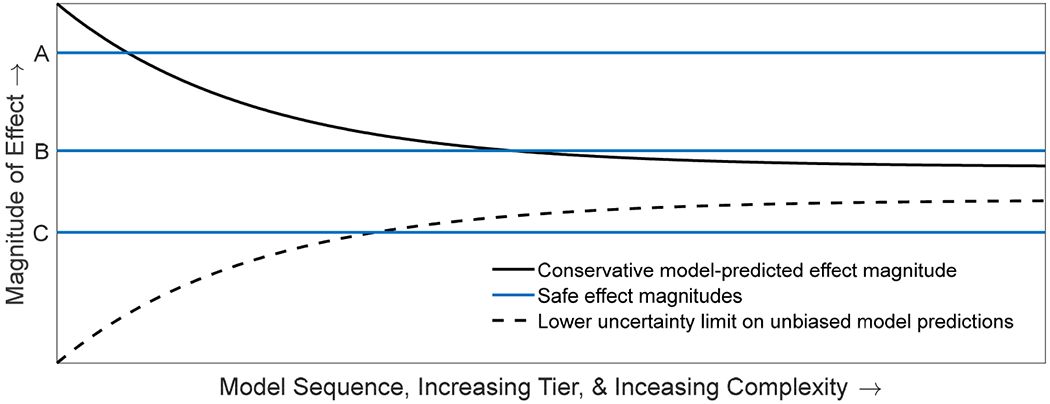
Conceptual model for three different risk assessments illustrating the efficiency principle and best modeling practices. The bold black curve is a conceptual model for *positively biased* model predictions of effect magnitude (vertical axis) that satisfy the efficiency principle with increasing complexity and tier (horizontal axis). Lines A, B, and C represent different risk assessments, for which an a priori ‘safe’ effect magnitude has been specified. The dashed curve represents the lower uncertainty bound for *unbiased* predictions of effect magnitude from models developed according to best modeling practices. When combined with the bold black curve (repurposed to represent the upper uncertainty bound for unbiased model predictions), the two form a hyperbolic cone representing declining uncertainty of unbiased model predictions developed using best practices. The ‘true’ (unknown, and so not pictured) effect magnitude is assumed to lie at the unrealized asymptote approached by both curves.

In Figure 1, horizontal lines represent a priori levels of effect determined to be safe/acceptable, which are independent of tier, model, and complexity. The monotonic decline in the positive bias of predicted effect magnitude under the efficiency principle ensures that a higher tier model with a smaller positive bias cannot overturn a ‘safe’ determination made at a lower tier (i.e., once the predicted effect magnitude curve crosses a safe threshold it will not cross back at a higher tier). Line A represents a risk scenario that could be determined acceptable with an easily parameterized lower tier model, such as a risk quotient because even a highly positively biased predicted effect magnitude is below line A. Line B represents a risk scenario in which the predicted effect magnitude is not revealed to be safe until a higher tier model is used. Line C represents a risk scenario that should never be determined safe because the true magnitude of effect (asymptote of the hyperbolic cone) is higher than the pre-determined acceptable effect magnitude.

When models are optimized according to best practices, their predictions will (ideally) be unbiased and so will vary both positively and negatively around the true magnitude of effect due to uncertainty and sampling error, and this uncertainty will decline at higher tiers (the hyperbolic cone depicted in Figure 1). With an unbiased sequence, a no/low-risk determination at a lower tier guarantees neither safety nor a consistent prediction at a higher tier. Importantly there is a region within which the unsafe scenario might be deemed safe when conservatism is not intentionally designed into the model sequence—the region below line C and above the dashed curve. This possibility (whether realized or not) may invalidate a no-risk determination as a stopping rule. In contrast, with an unbiased model sequence, there is a greater possibility of making a no/low-risk determination at lower tiers, but this determination would not carry the same level of confidence as if it were made under the efficiency principle because a higher tier model might predict greater effect magnitudes, thereby overturning the risk conclusion.

Embracing the efficiency principle leads to a difficult dilemma. On the one hand, the development of a series of increasingly realistic models that produce reliably diminishing conservative bias in predictions presumes foreknowledge of model predictions and bias along the series and a complete understanding of the effect (in the model) of introducing added realism. On the other hand, if we are not confident in the inverse relationship between conservative bias and realism, then an alternative set of decision criteria for stopping versus escalating must be articulated. Criteria that focus on optimizing model design commensurate with the objectives of a risk assessment and the available data (i.e., best practices) [3,4,17,21] Schmolke et al. (2017), Raimondo et al. (2018, 2020) are a natural alternative. However, such criteria may leave risk assessments vulnerable to the criticism that more complexity and realism might overturn the risk conclusion.

Many additional factors conspire against our ability to develop a parsimonious sequence of conservative models. Foremost among these is that model endpoints are not comparable across tiers. For example, is RQ = 1.5 more or less risky than Δλ = 0.05? This question is, at best, difficult to answer and, at worst, meaningless. It highlights two issues that are not accommodated well by the conceptual model above—that effect magnitudes are expressed in different units at different tiers and that they are measured on different scales. But there are other, more mundane considerations as well. Given the resource constraints involved in model development for ecological risk assessment, existing models may be pressed into service in ways not originally anticipated. For example, consider two hypothetical models, Model A and Model B. Model A may be more conservative under some parameterizations, whereas model B may be under others. To which tier(s) do we assign the two models? Even worse, what if the rank-reversal occurs within the parameter space under consideration in the risk assessment?

### 3.2. Evaluation of a Model Escalation Sequence

#### 3.2.1. Risk Quotients → MCnest

Acute and chronic RQs for 13 pesticides are given in Table 2 [16]. Exposure estimates for RQs were generated using the Terrestrial Residue EXposure Model [18], and effects estimates were taken from studies submitted to the USEPA. Of those 13 pesticides, 7 had acute or chronic RQs that exceeded LOCs and were chosen for higher tier modeling using MCnest. Consistent with EPA guidance [18], RQs were generated with the lowest available toxicity endpoints from any study considered scientifically valid and reliable as a quantitative estimate of toxicity. For MCnest modeling, toxicity endpoints were limited to those generated from mallard (Anas platyrhynchos) or northern bobwhite (Colinus virginianus) to standardize interspecies extrapolations to the greatest extent possible. MCnest simulations employed the Terrestrial Investigation Model (TIM) [30] to generate exposure and adult mortality estimates. Additional realism conferred by the use of MCnest compared to RQs included treatment of exposures as a distribution, rather than a single upper bound value, treatment of diet as a mixture of components (e.g., invertebrates, seeds, etc.) with different pesticide residues, and binomial modeling of foraging on and off-field. The objective of the study was to evaluate the relative risk, among the 13 original pesticides, to birds using agroecosystems, and absolute risk estimation was not attempted.

#### 3.2.2. Why Might RQs Be More Conservative than MCnest?

RQs, as calculated in [16], compare upper bound exposure to a toxicity endpoint regardless of the timing of exposure. For example, birds experiencing exposures exceeding reproductive NOAELs outside of the breeding season might not experience any adverse effects if those exposures are also well below acute thresholds. Further, if the bird is migratory, individuals may not experience any exposure at all. MCnest takes the timing of exposure into account by modeling initial pesticide concentrations in the environment following application and models the decay of the pesticide according to its degradation half-life. Therefore, considering the timing of exposure using MCnest is an increase in realism achieved by relaxing the conservative assumption of static exposure compared to deterministic RQs. In the example cited above [16], pesticide applications were associated with specific dates based on labeling requirements for the pesticides, and the timing of avian breeding was based on literature reports for the modeled species in the modeled system (upper Midwest agricultural ecosystems).

Although MCnest also uses threshold comparisons to determine whether a nest fails or succeeds, birds may compensate for a lost attempt by renesting if time remains in their modeled breeding season. This approach is also less conservative than a static RQ. Further, many, though not all, MCnest surrogate endpoints use time-weighted averages of exposure from the modeled decay curve, with time > 1 day, so that the values of the numerator in the MCnest exposure/toxicity comparisons would be lower than peak exposure even on application day. Finally, eliminating studies on species other than northern bobwhite and mallard during MCnest modeling but including them for RQs, meant that some of the toxicity endpoints used in RQs were lower than the corresponding values used in MCnest.

#### 3.2.3. Why Might MCnest Be More Conservative than RQs?

MCnest simulations [16] were conducted using the Terrestrial Investigation Model to generate exposure estimates. The choice to do so follows the expected increase in realism with tier escalation, as TIM includes many realistic processes not included in T-REX. For example, TIM includes first-order elimination kinetics when calculating avian dose, and it includes additional exposure pathways such as dermal exposure, drinking water, and inhalation. This added realism could introduce conservatism. If elimination is slow, then the internal dose could exceed external exposure (daily dose based on environmental concentrations using the T-REX method). Similarly, if inhalation, drinking, or dermal exposure are important pathways, then the calculated total dose could exceed the dietary dose that was used for T-REX RQ calculations.

To evaluate the extent to which this may have occurred, a limited set of simulations were rerun in MCnest for three insectivorous songbirds, tree swallow (Tachycineta bicolor), house wren (Troglodytes aedon), and black-capped chickadee (Poecile atricapillus). Table 3 presents the differences in MCnest predictions with TIM versus T-REX, where negative values indicate that MCnest with TIM offered more conservative predictions and vice versa. In general, MCnest with TIM generated less conservative predictions than MCnest with T-REX, but this was not universally true with the three re-analyzed species and seven pesticides.

#### 3.2.4. MCnest → ELM

Etterson and Ankley [24] used MCnest output as input for an ELM that modeled aryl hydrocarbon receptor (AHR) activation, leading to reproductive effects in two bird species, tree swallow and bald eagle (Haliaeetus leucocephalus). The species were chosen to represent a long-lived bird with delayed sexual maturation (bald eagle, first reproduction at year 6) compared to a short-lived bird that begins reproduction at 1 year (tree swallow). The purpose of that work was to demonstrate the ability of ELMs to integrate toxicological effects to predict fitness effects, taking lifecycle into account.

Table 4 reports the magnitude of effects on MCnest predictions versus ELM predictions for embryonic mortality associated with AHR activation at the LC50. For bald eagle, the effects on fitness are much larger than effects on fecundity, whereas, for tree swallow, the effects on fitness are much smaller than effects on fecundity. On its face, this appears to be a potential case of the hypothetical Model A/Model B scenario presented above. However, caution is warranted. Model predictions are not similarly scaled, and proportional reductions tell a different story. For both species, annual fecundity (MCnest prediction) and lifetime reproductive success (ELM prediction) are reduced by 50% compared to the same metrics in the absence of AHR activation. Intrinsic fitness (ELM prediction) is reduced by 33% for tree swallow and only 6% for bald eagle, again relative to expected values in the absence of AHR receptor activation. Thus, from a proportional reduction perspective, the models are either equally conservative (comparing MCnest predictions to lifetime reproductive success) or the ELM is less conservative (comparing MCnest predictions to intrinsic fitness) for both species.

The above discussion highlights the difficulty we face in implementing the efficiency principle in an escalating model sequence. However, the interpretational challenge is not limited to proportional versus absolute effects. In the preceding paragraph, a diminishing proportional difference between model predictions in the exposed versus the control scenario was used as a proxy for a decline in conservative bias when comparing MCnest predictions to intrinsic fitness. Strictly speaking, that argument requires that control predictions for both MCnest and ELM are unbiased. However, if both control and exposed scenarios in an ELM are highly negatively biased, then the proportional difference might decline between MCnest and an ELM, while at the same time, ELM predictions could have higher “conservative” bias than MCnest. This highlights our greatest challenge in implementing the efficiency principle: without knowing the true risk, we cannot know model bias.

#### 3.2.5. Why Might MCnest Be More Conservative than an ELM?

The argument presented above suggests that effects on fecundity will result in proportionally similar or proportionally smaller reductions in fitness in ELM predictions compared to MCnest predictions, depending on the output metric employed. Therefore, assuming the control predictions are unbiased for both MCnest and ELM, the conservative bias inherent in ELM lifetime reproductive success predictions would be less than the conservative bias in fecundity predictions from MCnest. Like the comparison from static RQs to MCnest, the step from MCnest to ELM increases realism and relaxes conservative bias by considering exposure in the context of a longer period of the lifecycle, a year (λ_f_) or a lifetime (LRS).

#### 3.2.6. Why Might an ELM Be More Conservative than MCnest?

When exposure induces effects on multiple vital rates, an ELM offers the simplest integration of effects that takes into account the species lifecycle. If exposure causes both acute and chronic effects, then an ELM will likely predict greater proportional effects than MCnest alone. In this case, greater realism is associated with greater conservatism. This reversal might cascade down to RQs (i.e., ELM more conservative than lowest RQ) if acute and chronic RQs are both greater than their respective LOCs [16].

#### 3.2.7. ELM → SEPM

The spatially explicit population model for California gnatcatchers [25] included habitat-specific determination of background vital rates, carrying capacity, and explicit movement rules. Each of these processes represents significantly increased realism compared to an ELM, which predicts only individual fitness. Below, simple ELM calculations are made using data from [24] for comparison with the gnatcatcher SEPM.

#### 3.2.8. Why Might an ELM Be More Conservative than a SEPM?

Table 5 gives background demographic rates for the gnatcatcher (in ideal habitat in the absence of exposure) [24]. Plugging those values into the ELM fitness equations (Equations (2) and (3)) gives an estimate for lifetime reproductive success (LRS) of 2.0312 female offspring produced on average in a lifetime. Similarly, the model gives an estimate of the annual propagation of female genetic descendants (λ_f_ of 1.495. Technically, these fitness measures are smaller than those that would be generated following the recommendations of [24] because the fecundity values are for female offspring only [25], in keeping with traditional population modeling practice. For this illustration, the distinction does not matter.

Under the greatest reduction in reproductive success predicted by MCnest for the reproductive stressor, f ≈ 0.65 (Figure 3 in [25]). Substituting f ≡ 0.65 for that reported in Table 5 and plugging all three values into the ELM equations (Equations (2) and (3)) gives λ_f_ = 0.8 and LRS = 0.4852. Neither of these values are sufficient fitness for a female to replace herself, either annually or during her lifetime, suggesting that a population of individuals experiencing identical conditions would likely decline to extinction. In contrast, the SEPM predicted persistence for at least 50 years. An analogous argument could be made with the survival stressor [25]. However, the inclusion of refugia (areas in which pesticides were not used) resulted in the added realism of the SEPM, relaxing the conservative assumption inherent in the ELM prediction, which pertained only to exposed individuals.

#### 3.2.9. Why Might an SEPM Be More Conservative than an ELM?

Like most SEPMs, the gnatcatcher model included density-dependence induced by movement limitation and patch-specific carrying capacities. When average fitness exceeds that required for a population to persist, then fitness calculated from an ELM will necessarily be higher than the same metric calculated from an SEPM at equilibrium. In that case, the SEPM would report the very minimum value of fitness required for persistence, whereas the ELM would report a value that is, by definition, higher. Modifications of the way in which fitness is calculated could be made to avoid this reversal in the magnitude of fitness (or reductions in fitness), but these would require foreknowledge of the effect of increased realism on the model predictions. In this simple example, this foreknowledge is relatively obvious, but in many cases, it would not be.

### 3.3. General Discussion and Recommendations

The comparisons above show that increased realism does not necessarily confer a reduction in conservative bias with tier escalation, even when the added realism is intended to relax conservative assumptions made in the preceding step. For each of the three escalation steps, it was shown that increased realism could either increase or decrease conservatism and that this is due to multiple considerations that would be in competition with one another to produce the actual relationship between realism and bias with tier escalation. It was further shown that these relationships are context-dependent and that it would be difficult, in any given application, to know a priori whether the efficiency principle is satisfied. Nested model sequences like those reviewed herein (i.e., output from tier n-1 as input for tier n) are helpful but not sufficient. These conclusions were reached using a specific suite of avian models, but the conclusions themselves likely apply very broadly to other model sequences that might be used in PLRA. The desiderata of risk assessment off-ramps to be achieved by “passing” some tier of a sequence of decreasingly conservative and increasingly realistic models will be difficult to achieve.

Best practices for developing models [2–4,17,21] will help produce more accurate models with increasing realism, but these will not necessarily satisfy the efficiency principle (Figure 1). First, the criterion of model accuracy is in direct conflict with the desire for conservative predictions, and best principles are just as likely to produce models that underestimate effects. Second, at some unknown point, increasing model realism exceeds the support of the data, and bias is likely to increase with complexity. The latter point is especially true when overparameterized models are applied to novel data. This again highlights the need for parsimony in identifying ideal model complexity for ecological risk assessment [21].

It should also be noted that alternative model parameterizations will also affect the performance of a model compared to the models that precede and succeed it in a risk assessment sequence. Life history traits vary widely among even closely related species and have the ability to influence the degree to which a model is conservative. For example, Table 3 contains inconsistencies in the relative conservatism of T-REX versus TIM parameterized for three insectivorous cavity-nesting passerines, species that should otherwise be very similar to one another. Other specifics of the risk assessment context will also likely influence relative model predictions such as the mode of action or adverse outcome pathway induced by exposure and the landscape setting in which exposure occurs.

The above exercises also offer some cause for optimism respecting the efficiency principle. Of the reviewed applications, only one [16] attempted to decrease conservatism with tier escalation, and, with a few exceptions, they appear to have been successful (see, e.g., Table 3). As argued above, model repurposing is likely to be the rule as we grow our PLRA toolboxes, giving us a suite of models with unknown bias and with unknown relationships to one another. Yet we may be able to use simulated data on well-studied chemicals in which risk is determined in advance to study the model sequence(s). When models are nested, as envisioned here, their properties and predictions will be more comparable.

From these arguments, several strategies for studying model escalation sequences suggest themselves. One strategy would be to simulate data using the highest-tier model and then evaluate the performance of each nested model on the simulated data. Many transitions would still be difficult, for example, the “field to lab” comparison, which would be the MCnest → RQ step in the inverted sequence from above. Another valuable strategy in deploying model escalation sequences might be to develop paired model parameterizations within tiers. For example, RQs could be generated with median exposure estimates and with upper-bound estimates as a way of gauging the effect of conservative assumptions on RQ predictions. Similarly, MCnest, or any other ecological model, could be run with and without conservative assumptions, keeping all other parameters fixed, to compare the impact of those assumptions on model predictions. Ideally, if the efficiency principle were implemented successfully, the distance between the median versus conservative predictions would diminish with tier escalation, though this might require rescaling the effect magnitudes to be similar among tiers. Finally, introducing conservativism through alternative parameterizations rather than alternative model structures will facilitate both the study of and implementation of efficiency in tiered risk assessment.

Hybrid approaches that employ both conservatism and best practices should be considered with caution. From the above, it is not clear that the two strategies are consistent with each other. Even if they can be reconciled, then a hybrid approach seems unlikely to realize either the benefit of efficiency (safe and conservative stopping rule) or best practices (accurate predictions commensurate with data). At a given tier, either one or the other strategy should be chosen. Thus, one possible hybrid approach might be to switch strategies at some point, relying on the efficiency principle at lower tiers and switching to best practices at higher tiers. This overall strategy could take advantage of the benefits of each at the tiers at which they would be most useful (efficiency at lower tiers, accuracy and realism at higher tiers). In any case, at the very highest tier, the efficiency principle will not be useful when the risk conclusion at that point is “not acceptable”.

Escalation of realism and complexity in model sequences will often be more complicated than represented here with a sequence of nested models. For example, an ELM could be much simpler than MCnest, incorporating only three or four parameters, though ELMs have been presented here as representing greater realism and complexity than MCnest. This was guaranteed in the above sequence by considering models as a nested sequence (i.e., with MCnest fecundity predictions as input to ELMs and ELM predictions considered as input to the SEPM). In practice, different model components may be more or less realistic or complex, depending on circumstances. For example, a complex and realistic exposure model may be implemented with effects models and/or life history models that are considered less realistic [3]. Similarly, model complexity and realism necessarily involve both model structure and parameterization so that a given model may be simplified by constraining parameter values (for example, by setting regression coefficients to zero), which again highlights the utility of nested models in an escalation sequence.

Successful implementation of the efficiency principle articulated above would help conserve resources for population-level ecological risk assessment when deploying a series of ever more realistic models. However, it may also be an ideal that cannot be perfectly achieved. Recent research has provided valuable momentum for the development of ecological models [3,4,17,21]. It is not too soon to put careful thought into how we will deploy and interpret them.

## Figures and Tables

**Table 1.** Example model escalation sequence for avian population level risk assessment arranged from lowest (RQ) to highest (SEPM) complexity.

Model	Prediction(s)	Citations
Risk Quotient (RQ)	Exposure/Toxicity	[16,19]
Markov Chain Nest Productivity Model (MCnest)	Annual reproductive success	[22,23]
Endogenous Lifecycle Model (ELM)	Intrinsic fitness and lifetime reproductive success	[24]
Spatially explicit population model (SEPM)	Population growth rate and population size	[25]

**Table 2.** Acute and chronic risk quotients (RQ = exposure/toxicity) for insectivores for 13 pesticides. The median lethal dose (LD50) units = mg active ingredient/kg bodyweight. The median lethal concentration (LC50) units = mg active ingredient/kg diet.

Pesticide	LD50	LC50	Test Species	Acute RQ	Chronic RQ
bifenthrin	1800	75	*Colinus virginianus*	0.0083	0.13
carbaryl	2290	300	*Coturnix japonica*	0.34	0.041
chlorantraniliprole	>2250	120	*Colinus virginianus*	<0.011	0.0031
chlorpyrifos	8.41	25	*Phasianus colchicus*	30	4.5
cyfluthrin	>2000	250	*Colinus virginianus*	0.011	0.39
dimethoate	5.4	4.0	*Agelaius phoeniceus*	12	12
esfenvalerate	381	608	*Colinus virginianus*	0.072	0.028
indoxacarb	98	144	*Colinus virginianus*	0.34	0.15
lambda-cyhalothrin	3950	5	*Anas platyrhynchos*	0.0057	2.1
malathion	167	110	*Phasianus colchicus*	1.8	0.039
methomyl	15	150	*Phasianus colchicus*	18	1.1
methoxyfenozide	>2250	819	*Colinus virginianus*	<0.057	0.014
permethrin	>9869	125	*Anas platyrhynchos*	<0.022	0.0099

**Table 3.** Differences in Markov Chain Nest Productivity Model (MCnest) fecundity predictions using the Terrestrial Residue EXposure (T-REX) model versus the Terrestrial Investigation Model (TIM) (negative values occur when fecundity predictions using T-REX exceed those using TIM for the same chemical use scenario).

Chemical	Tree Swallow	House Wren	Black-Capped Chickadee
None	0	0	0
Carbaryl	−0.01	3.78	4.40
Chlorpyrifos	0.13	−2.08	0.24
Indoxacarb	1.53	3.51	4.21
Lambda cyhalothrin	3.13	3.66	0.43
Malathion	−0.21	1.09	0.22
Methomyl	0.21	−1.49	0.46
Permethrin	3.78	3.81	1.30

**Table 4.** Markov Chain Nest Productivity Model (MCnest) predictions compared to endogenous lifecycle model (ELM) predictions for aryl hydrocarbon receptor (AHR) activation leading to embryonic mortality at the median lethal concentration (LC50).

Model	Prediction	Bald Eagle	Tree Swallow
MCnest	Annual Reproductive Success	0.7	1.91
ELM	Lifetime Reproductive Success (LRS, Equation (2))	1.44	0.78
ELM	Intrinsic Fitness (λ_f_, Equation (3))	1.01	0.88

**Table 5.** California gnatcatcher vital rates in ideal habitat.

Rate	Value	Definition
s_j_	0.4314	survival from fledging to recruitment at age 1
s_a_	0.5200	annual adult survival
f	2.2600	annual reproductive success

## Data Availability

Data used in this article are publicly available through the original publications cited in this review.
